# Changes in Brain Responses to Music and Non-music Sounds Following Creativity Training Within the “Different Hearing” Program

**DOI:** 10.3389/fnins.2021.703620

**Published:** 2021-10-01

**Authors:** Anna Arkhipova, Pavel Hok, Jan Valošek, Markéta Trnečková, Gabriela Všetičková, Gabriela Coufalová, Jaromír Synek, Vít Zouhar, Petr Hluštík

**Affiliations:** ^1^Department of Neurology, Faculty of Medicine and Dentistry and University Hospital Olomouc, Olomouc, Czechia; ^2^Department of Biomedical Engineering, University Hospital Olomouc, Olomouc, Czechia; ^3^Department of Computer Science, Faculty of Science, Palacký University Olomouc, Olomouc, Czechia; ^4^Department of Music Education, Faculty of Education, Palacký University Olomouc, Olomouc, Czechia

**Keywords:** creativity, brain plasticity, music training, task-related fMRI, auditory perception, music composition, music education

## Abstract

The “Different Hearing” program (DHP) is an educational activity aimed at stimulating musical creativity of children and adults by group composing in the classroom, alternative to the mainstream model of music education in Czechia. Composing in the classroom in the DHP context does not use traditional musical instruments or notation, instead, the participants use their bodies, sounds originating from common objects as well as environmental sounds as the “elements” for music composition by the participants’ team, with the teacher initiating and then participating and coordinating the creative process, which ends with writing down a graphical score and then performing the composition in front of an audience. The DHP methodology works with a wide definition of musical composition. We hypothesized that the DHP short-term (2 days) intense workshop would induce changes in subjective appreciation of different classes of music and sound (including typical samples of music composed in the DHP course), as well as plastic changes of the brain systems engaged in creative thinking and music perception, in their response to diverse auditory stimuli. In our study, 22 healthy university students participated in the workshop over 2 days and underwent fMRI examinations before and after the workshop, meanwhile 24 students were also scanned twice as a control group. During fMRI, each subject was listening to musical and non-musical sound samples, indicating their esthetic impression with a button press after each sample. As a result, participants’ favorable feelings toward non-musical sound samples were significantly increased only in the active group. fMRI data analyzed using ANOVA with *post hoc* ROI analysis showed significant group-by-time interaction (opposing trends in the two groups) in the bilateral posterior cingulate cortex/precuneus, which are functional hubs of the default mode network (DMN) and in parts of the executive, motor, and auditory networks. The findings suggest that DHP training modified the behavioral and brain response to diverse sound samples, differentially changing the engagement of functional networks known to be related to creative thinking, namely, increasing DMN activation and decreasing activation of the executive network.

## Introduction

Creativity is one of the essential human-specific constructs and it has been consensually defined as an ability to produce novel and useful/appropriate/valuable ideas/works, both in the general public and among researchers (Runco and Jaeger, 2012). Creativity applies not only to specific domains such as music, visual arts, sciences, and industry, but also to many details of daily work and life, thus, high creativity has a big impact on the society and quality of many scenes in human life. Domain-general creativity can be enhanced by training of a certain modality, such as musical creativity, embedded in the process of composing. Many research efforts focus on specialized music teaching and further development of musical abilities (Hickey, 2002; Lapidaki, 2007; Running, 2008; Webster, 2016). In our research, we focus on the development of musical creativity through group compositional techniques in children and students. Against the background of the many proposed definitions of musical creativity (see, e.g., Cook, 2011; Burnard, 2012; Hargreaves, 2012), our work defines creativity operationally as the ability to include non-musical sounds and silence to make music, to engage in group composing, yielding a concrete result (composition), subsequently presented in public and providing satisfaction to participants, teachers/instructors as well as to audience (which was not part of the composing process).

The “Different Hearing” program (DHP) is a musical cognitive training aimed at stimulating creativity by means of group music composing in the classroom, using objects from everyday life rather than traditional musical instruments, employing non-musical sounds (Zouhar and Medek, 2010; Coufalová and Synek, 2014). The program was established in 2001 at the Department of Music Education of Palacký University Olomouc, Czechia, as an alternative method to the mainstream model of music education. The DHP methodology is based on Cage’s (1973) wide meaning of musical composition: “The material of music is sound and silence. Integrating these is composing.” (p. 62). Participants in the DHP workshop gain both knowledge that all kinds of sounds can be used and put together to create music and the practical skills how to do so. Group composing in the DHP is similar to the approach of composers and educators Paynter and Aston (1970), Schafer (1986), Schneider et al. (2000), or Laycock (2005). DHP has primarily focused on children and young people age five to eighteen, although young adults have been repeatedly studied as well (Zouhar and Medek, 2010; Coufalová and Synek, 2014). Detailed evaluations of a series of DHP workshops since 2002 are performed through structured questionnaires and clearly indicate a potential to enhance self-perceived musical creativity and change subjective appreciation of music and sounds (Synek, 2008; Medek et al., 2014). Changes of non-musical sound perception could be illustrated by a participant’s statement: “Začala jsem více vnímat zvuky (v hlučném městě zpěv ptáků)./I started paying more attention to sounds (birds singing in the noisy town)” (Synek, 2008, p. 119).

Although we have not found published evidence for neuroanatomical correlates of behavioral changes after musical creativity training, functional neuroimaging studies of domain-general creativity suggest association/relationship between the creative performance and brain function.

For example, functional neuroimaging studies in creativity suggest that there is a correlation between domain-general creativity and functional connectivity within well-defined resting state networks, namely, in the default mode network (DMN) and executive-control network (EN) as well as the salience network (Beaty et al., 2017, 2018). Observational studies of creative performance, however, identify only the brain structures associated with static creative traits. Further insight may be gained by evaluating dynamic processes, such as increasing one’s capacity for creative performance. For example, in a fMRI study by Fink et al. (2015), activation changes after 3-week domain-general creativity training (20 min/day) were observed in the left inferior parietal lobule (IPL) and the left middle temporal gyrus (MTG). Also, Sun et al. (2016) observed change in brain function in the dorsal anterior cingulate cortex (dACC) and dorsolateral prefrontal cortex (DLPFC) as well as increased gray matter volume in dACC after 20 sessions (28 min/day) of creativity training. Successful training in musical creativity would be similarly expected to induce functional plasticity of the participating brain systems. Training-induced changes may subsequently be observed not only during creative activity, but also during perception of different sound classes. This approach (using a different task to probe the brain networks changed by training or stimulation) has been used in other plasticity-inducing protocols, including but not limited to music training (Schlegel et al., 2015; Tierney et al., 2015; Herholz et al., 2016; Sachs et al., 2017).

Since DHP has a potential to develop and/or enhance musical creativity and change subjective appreciation of music and sounds (Synek, 2008; Medek et al., 2014), we hypothesized that a 2-day intense workshop would induce changes in subjective appreciation of different classes of music and sound (including typical DHP samples), as well as plastic changes of the brain systems engaged in creativity and music perception, in response to diverse auditory stimuli.

To address these hypotheses, we designed a randomized behavioral and fMRI study, where an active group would be scanned twice, before and after participating in the DHP workshop, and a control group would also be scanned twice within the same time interval. FMRI has been repeatedly used to describe neuroplastic changes related to behavioral training and learning. The subsequent analysis was designed to evaluate the effect of the intervention either (1) with respect to the perception of different sample classes or (2) regardless of the sample class.

## Materials and Methods

### Subjects

**FIGURE 1:**
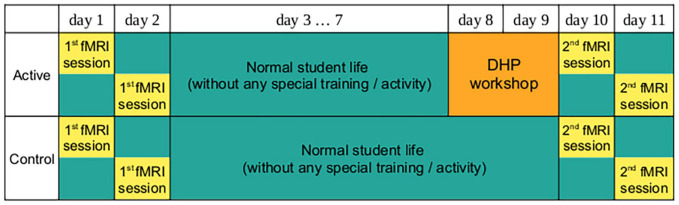
Flow Chart of the Study. All subjects had a same-length interval (8 days) between the first and second MRI measurements. The second examination of the active group was performed within 2 days after the DHP workshop.

Forty-six healthy volunteers with normal hearing and with no history of neurological disorders participated in this study (40 females and 6 males, mean age 21.6 ± 1.4). The subjects were Teacher Training for Primary Schools students from the Faculty of Education at Palacký University Olomouc, who had 10.3 ± 5.0 years of music education. Four participants were left-handed, two were ambidextrous, and 41 were right-handed as assessed by the Edinburgh handedness inventory (Oldfield, 1971). The study was carried out in accordance with World Medical Association Declaration of Helsinki. Written informed consent was obtained from all participants prior to their inclusion in the study and the study was approved by the Ethics Committee of the Faculty of Education at Palacký University in Olomouc, approval number 02/2017. Twenty-two participants were randomized to participate in the DHP workshop with the length of 10.5 h over two consecutive days (active group) and 24 students were randomized to the control group to receive no special training and continued normal daily activities and student life. Both groups underwent two fMRI examinations 8 days apart - for the active group, one was before and one after the workshop (Figure 1). Both groups were matched in terms of music education, according to computed results from self-reported questionnaires (unpaired t-test on 7 items scoring music education, all p-values > 0.05).

### Description of the “Different Hearing” Program Workshop

DHP aims at stimulating and enhancing children’s own creativity by means of learning group music composing in the classroom. As already mentioned in the Introduction, participants in the DHP workshop gain both knowledge that all kinds of sounds can be used and put together to create music and the practical skills how to do so (see below). The program was established in 2001 at the Department of Music Education of Palacký University Olomouc, Czechia, as an alternative method to the mainstream model of music education. The DHP methodology is based on Cage’s (1973) wide meaning of musical composition: “The material of music is sound and silence. Integrating these is composing.” (p. 62). Group composing activities are oriented similar to composers and educators Paynter and Aston (1970), Schafer (1986), Schneider et al. (2000), or Laycock (2005). It has primarily focused on children and young people age five to eighteen, although young adults have been repeatedly studied as well (Zouhar and Medek, 2010; Coufalová and Synek, 2014).

In our study, the length of the DHP workshop was 10.5 h, divided over two consecutive days, and the instructors functioned as partners/co-performers initiating and then participating and coordinating the creative process, rather than acting as teachers. On the first day, participants were trained to discover a new sonic world, namely by creating sounds using their own bodies/voices, and by creating original musical instruments made of materials from everyday life such as plastic bottles, wooden chairs, recorded ambient sounds from the streets, etc., but without using traditional musical instruments in the traditional way. Next, they learned basic principles of improvisation and composition, as well as creation of their own graphic notations using original symbols. On the second day, the participants were divided into three groups and composed music using their original sounds and prepared the graphic scores, then performed the compositions in front of the other groups and instructors, followed by a discussion for evaluations and feedback.

### fMRI Task

Each fMRI examination included two functional imaging acquisitions during the task of listening to musical and non-musical sound samples from five different classes, i.e., Classic music, New music, DHP samples (composed and performed by previous participants during the workshops in the past), Nature sounds, and Industrial noises. Classical music and New music samples were extracted from musical recordings (CD tracks). The Classical music samples cover the classes from Middle Ages (Leonin, Machaut) and Baroque music (Monteverdi, Handel, Vivaldi), through Classical Period (Gluck, Stamitz, Beethoven) to Romantic music (Chopin, Verdi, Bruckner). The selection of New music spans a wide range of 20th century esthetics from Schoenberg, Hába, Cowell, Varèse to Boulez, Stockhausen, Xenakis, Ligeti, Lutosławski and Štědroň. The samples were chosen according to various parameters – in the classical music group, compositions from different stylistic periods (Middle Ages – 20^th^ century) were chosen in such a way that vocal, instrumental and vocal-instrumental, chamber and large-scale compositions were represented. Samples from the New music section represent different styles and various sound qualities related to contemporary music with the aim to provide a colorful selection of sounds. DHP sound compositions samples were cut from tracks recorded during different former workshops. Nature sounds and industrial sounds are field recordings – in the city, nature, etc., by members of the DHP team.

For each of these sound classes, 12 unique samples 15 s long were prepared. During each imaging run, fifteen different samples were played through MR-compatible headphones in a counterbalanced order across subjects, such that three different samples from each sound class were presented in each run. Each session consisted of two such imaging runs with different sound samples, thus, no sample was repeated twice for any subject. The volume level of sounds was adjusted in the audio editing software Audacity^1^ to be equal across all the samples. Furthermore, participants were asked to keep their eyes open and watch a fixation cross during the listening phase. They were instructed to press one of two buttons (like/not like) after listening to each sample as soon as the question “Did you like the sample?” (in Czech) appeared on screen (displayed for 4 s following the sound sample offset).

### MRI Data Acquisition

MRI examinations were performed twice with a 8-day interval for all subjects (the second examination of the active group was scheduled within 2 days after the workshop), using a 3T scanner (Siemens Prisma, Erlangen, Germany) with a standard 64-channel head and neck coil in the Multimodal and Functional Imaging Laboratory (MAFIL), Central European Institute of Technology (CEITEC) in Brno. The subject’s head was immobilized with cushions to assure maximum comfort and minimize head motion. The MRI protocol included task-related blood oxygenation level-dependent (BOLD) fMRI data acquisition (T2^∗^-weighted echo-planner imaging; 48 slices, 3mm slice thickness; repetition time/echo time = 780/35ms; flip angle 45°; field of view = 192mm; matrix 64 × 64; 465 volumes; repeated twice) during listening to the sound samples through MR-compatible headphones. Gradient-echo phase and magnitude fieldmap images were acquired to allow correction of the echo planar imaging distortions. A high resolution T1-weighted structural image was acquired using Magnetization-Prepared rapid Gradient-Echo (MPRAGE) sequence for anatomical reference. In addition, resting-state fMRI with BOLD EPI imaging data were obtained before the task-related acquisition, but the data is not reported here. Heart rate (pulse oximetry) and respiration (respiratory belt) were monitored during BOLD scanning.

### BOLD MRI Data Pre-processing

The fMRI data were processed using FEAT Version 6.00, part of FSL (FMRIB’s Software Library), version 5.0.9 (Jenkinson et al., 2012). The fMRI data were initially checked for susceptibility artifacts and none of the subjects were excluded. The pre-processing consisted of: correction of B0 distortions, motion correction, non-brain tissue removal, and spatial smoothing using a Gaussian kernel with 8.0 mm full width at half maximum (FWHM). During pre-processing, an affine registration matrix between the functional images and the respective structural image was obtained using FLIRT (Jenkinson and Smith, 2001; Jenkinson et al., 2002) and a non-linear transformation between the structural space and the MNI 152 standard space was calculated using FNIRT (Grabner et al., 2006). Next, using partially pre-processed data, motion-related artifacts were regressed out from functional time-series by ICA-AROMA automatic noise classifier (Pruim et al., 2015), followed by high-pass temporal filtering with sigma = 60.0 s. Finally, pre-processing included estimation of nuisance signal regressors based on the RETROICOR method (Glover et al., 2000).

### Statistical Analysis of BOLD Imaging Data

Voxelwise general linear model (GLM) analysis was carried out using FILM (Woolrich et al., 2001). At the single-subject level, the GLM consisted of 5 regressors to separately model activation evoked by each sound type, 2 regressors to model positive and negative feedback responses, and a single regressor to model activation due to visual presentation of the instruction. Temporal derivatives of each regressor were added to account for non-uniform slice timing and haemodynamic response function (HRF) delay. Eight more nuisance regressors obtained from RETROICOR were added to account for physiological noise. Single-subject contrasts were set to provide mean activation for each sound type.

Next, average effects per subject were computed using a fixed-effects analysis. The resulting beta parameters and residuals were then carried over to the group-level mixed effects analysis. At the group level, an analysis of variance (ANOVA) with 3 factors (time, group, and sound type) accounting for repeated measures was employed to test the main hypotheses. Following F-tests were evaluated: (1) the goodness-of-fit of the ANOVA model (global F-test), (2) mean activation/deactivation, (3) group-by-time-by-sound type interaction, and (4) group-by-time interaction. The mixed-effects analysis was performed using FLAME (FMRIB’s Local Analysis of Mixed Effects) stage 1 (Beckmann et al., 2003; Woolrich et al., 2004). The Z (Gaussianised T) statistic images were thresholded using clusters determined by Z > 3 and family-wise error (FWE) corrected cluster significance threshold was *p* < 0.05 (Worsley, 2001). To assess the directionality of significant changes, a *post hoc* t-test was performed for the interaction where significant clusters were detected. Only voxels falling within significant clusters in F-test 1, 2, and 3 (for group-by-time-by-sound interaction) and F-tests 1, 2, and 4 (for group-by-time interaction) were considered. The *post hoc* analysis images were thresholded voxel wise at the FWE corrected *p* < 0.05. Significant clusters bigger than 100 voxels were anatomically classified according to an overlap with the Harvard-Oxford Cortical and Subcortical Structural Atlases (Desikan et al., 2006), and the Probabilistic Cerebellar Atlas labels (Diedrichsen et al., 2009). The resulting statistical images were rendered in Mango v4.0 (Research Imaging Institute, UT Health Science Center at San Antonio, TX, United States).

### Analysis of In-Scanner Behavioral Data

The effect of time/session on subjective like/not like response to individual stimulus classes was tested within subject using Wilcoxon signed rank tests in the active and control groups.

## Results

### Behavioral Data

Comparison of (behavioral) responses to sound samples showed that favorable feelings toward DHP, New music and non-musical sound samples (Nature and Industrial) significantly increased only in the active group (*p* < 0.05, Wilcoxon signed rank tests). The change for the DHP sound samples was most robust and survived optional Bonferroni correction for multiple testing. Descriptive statistics for the individual sound classes across groups and sessions is provided in Table 1.

**TABLE 1:** Behavioral responses to individual sound classes by group and session.

		**Session 1**			**Session 2**			
**Group**	**Sound class**	**Median**	**25%**	**75%**	**Median**	**25%**	**75%**	***P*-value**
**Active**	Classical	100.0%	100.0%	100.0%	100.0%	87.5%	100.0%	0.8359
	New	16.7%	0.0%	45.8%	50.0%	33.3%	66.7%	*0.0389*
	DHP	16.7%	16.7%	33.3%	50.0%	20.8%	66.7%	**0.0043**
	Nature	83.3%	66.7%	100.0%	100.0%	100.0%	100.0%	*0.0244*
	Industrial	33.3%	16.7%	55.4%	66.7%	33.3%	83.3%	*0.0397*
**Control**	Classical	100.0%	87.5%	100.0%	100.0%	100.0%	100.0%	*0.0479*
	New	50.0%	33.3%	66.7%	25.0%	16.7%	50.0%	0.1878
	DHP	50.0%	20.8%	66.7%	25.0%	16.7%	33.3%	0.1195
	Nature	100.0%	100.0%	100.0%	83.3%	72.9%	100.0%	0.0902
	Industrial	66.7%	33.3%	83.3%	33.3%	19.2%	50.0%	0.2651

*P-value: Wilcoxon signed-rank test (session 1 – session 2). Bonferroni-corrected threshold is 0.05/10 = 0.005. Statistically significant changes are displayed in **bold** (corrected) and italics (uncorrected). DHP, “Different Hearing” program.*

### Imaging Results

The main hypotheses of our study were tested within the framework of a comprehensive statistical model, including all the main effects (group, time, and sample class), interactions and specific *post hoc* contrasts. Firstly, the global F-test for the goodness-of-fit of the ANOVA model yielded significant clusters in a number of areas, including but not limited to the temporal and frontal cortices and cerebellum. Next, the highest level interaction (group-by-time-by-sound type, F-test 3) yielded an empty map (data not shown), meaning that there were no statistically significant clusters showing this particular effect. In other words, there was no significant difference among the interactions for each individual sample class.

**FIGURE 2:**
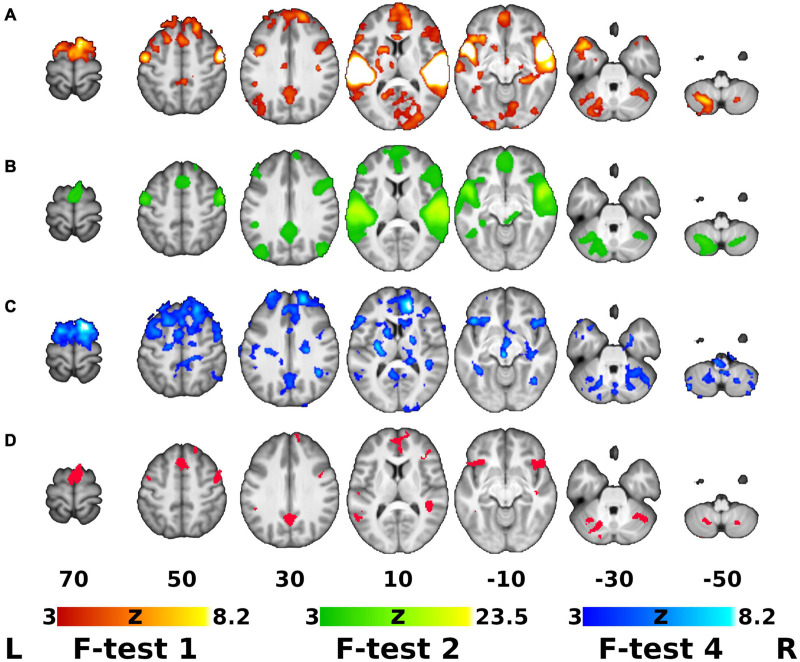
Global F-test of the ANOVA Model, Overall Activation/Deactivation, Group-by-Time Interaction and the ROI Mask for *Post hoc* Analysis. **(A–C)** Thresholded Z-score (normalized F-statistics) maps on top of an average T1-weighted structural image. **(A)** Global F-test of the analysis of variance (ANOVA) model (F-test 1, red-yellow overlay). **(B)** Mean activation/deactivation map across all sound classes (F-test 2, green overlay). **(C)** Areas showing differential effect of training/time between the groups (Group-by-time interaction, F-test 4, blue overlay). **(D)** Binary region of interest (ROI) mask resulting from conjunction of maps **A** ∩ **B** ∩ **C** (red). Maps **(A–C)** were cluster-wise thresholded using corrected cluster significance of *p* < 0.05 and cluster-forming threshold of Z > 3.0. Right is right according to neurological convention.

Our next hypothesis addressed the differential effect of time (training) between the active and control groups, which was captured by the group-by-time interaction (F-test 4). As described in the Methods, only voxels significant on the global F-test (F-test 1) and manifesting significant mean activation/deactivation effect (F-test 2) were considered further. Figure 2 shows the statistical parametric maps for (A) the overall effect (F-test 1), (B) mean activation (F-test 2), (C) group-by-time interaction (F-test 4) and (D) the region of interest (ROI) mask created by conjunction analysis of F-tests 1, 2, and 4.

**FIGURE 3:**
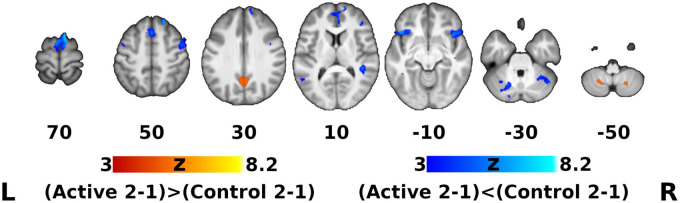
Thresholded Statistical Parametric Maps for *Post hoc* Contrasts within the ROI Mask (Figure 2D). Orange: Clusters manifesting significantly greater activation difference over time for the Active group compared to the Control group (posterior cingulate/precuneus, inferior cerebellum). Blue: Clusters manifesting significantly smaller activation difference over time for the Active group compared to the Control group (see Table 1 for the list of clusters). Other conventions same as Figure 2.

**FIGURE 4:**
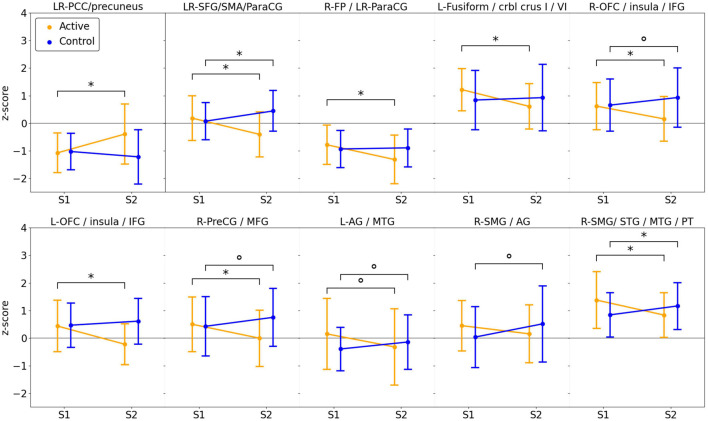
Line Graphs Demonstrating Average Activation for Each Group and Session within Each Cluster Displayed in Figure 3. Statistically significant differences between sessions (paired t-test) are shown by brackets and an asterisk (*), trends (0.05 < *P* < 0.1) are indicated by a circle (°).

The group-by-time interaction and subsequent *post hoc* analysis of fMRI data showed that, regardless of auditory sample class, activation in the active group apparently increased after DHP training (loss of deactivation) in the bilateral posterior cingulate cortex (PCC) and precuneus, which are functional hubs of the DMN. Moreover, significantly decreased activation in the active group was observed in the left inferior frontal gyrus (IFG), bilateral frontal orbital cortex (OFC) and right anterior cingulate cortex (ACC), which are parts of the EN, as well as in the regions in the motor network (bilateral supplementary motor cortex [SMA], right pre-supplementary motor area [pre-SMA] and left cerebellum) and auditory network (left MTG, right superior temporal gyrus [STG], planum temporale [PT] and temporal pole [TP]), whereas activation in all of these networks (EN, motor network, auditory network) in the control group was significantly increased (Figures 3, 4). For detailed description of the *post hoc* clusters, see Table 2.

**TABLE 2:** Significant group-by-time interaction – *Post hoc* analysis.

**Contrast**	**Cluster index**	**Volume [cm^3^]**	**Atlas label**	**Z_*max*_**	**Z_*max*_ Coordinates [x, y, z (mm)]**
(Active S2-S1) > (Control S2-S1)	1	2.79	31.2% L cingulate gyrus, posterior division 30.7% L precuneous cortex 25.8% R precuneous cortex 12.3% R cingulate gyrus, posterior division	7.06	−4, −54, 18
	1	10.40	35.5% R superior frontal gyrus 17.5% R SMA 16.2% L superior frontal gyrus 14.0% L SMA 11.1% R paracingulate gyrus 5.8% L paracingulate gyrus	9.01	10, 22, 66
	2	3.86	55.3% R frontal pole 21.3% R paracingulate gyrus 14.5% L paracingulate gyrus 6.8% R cingulate gyrus, anterior division	7.82	14, 58, 20
	3	3.20	51.0% L CRBL crus I 38.3% L occipital fusiform Gyrus 34.0% L CRBL VI 25.0% L temporal occipital fusiform cortex	7.01	−36, −80, −24
(Active S2-S1) < (Control S2-S1)	4	2.49	47.9% R frontal orbital cortex 23.2% R insular cortex 12.2% R inferior frontal gyrus, pars triangularis 9.6% R temporal pole	7.15	42, 16, −6
	5	2.23	65.6% L frontal orbital cortex 11.5% L insular cortex 11.5% L inferior frontal gyrus, pars opercularis 5.4% L frontal operculum cortex	8.37	−44, 20, −6
	6	1.51	79.4% R precentral gyrus 19.0% R middle frontal gyrus	5.66	50, 2, 48
	7	1.46	45.6% L angular gyrus 41.8% L middle temporal gyrus, temporooccipital part 7.7% L lateral occipital cortex, superior division	5.98	−48, −52, 2
	8	1.27	49.7% R supramarginal gyrus, posterior division 48.4% R angular gyrus	4.70	54, −48, 18
	9	1.08	40.0% R supramarginal gyrus, posterior division 23.7% R superior temporal gyrus, posterior division 17.8% R middle temporal gyrus, temporooccipital part 13.3% R planum temporale	6.39	44, −36, 8

*CRBL, cerebellum; L, left; R, right; S1, session 1; S2, session 2; SMA, supplementary motor cortex (Juxtapositional Lobule Cortex); Z_*max*_, maximum Z score.*

No *post hoc* analysis was performed for the three-way interaction as there were no significant clusters detected, as already mentioned.

## Discussion

### “Different Hearing” Program and Creativity

Before we proceed to the discussion of the study results, we will discuss in more detail the concept and design of the DHP and its relationship to creativity, as defined by our group.

The DHP was developed as an alternative music education approach. Similarly to other contemporary approaches (Hickey, 2002; Lapidaki, 2007; Running, 2008; Webster, 2016), DHP uses group composition in the classroom, encouraging the participants to move beyond traditional use of traditional music instruments to make music; instead, to include any non-musical sounds as well as silence in the course of group composing, organizing their work with the help of graphical scores and finishing with a concrete result (composition). From the cognitive neuroscience perspective, the workshop becomes a special case of musical cognitive training.

The new skills acquired during the DHP workshop represent a particular/specific type of musical creativity. In fact, Burnard (2012) pointed out that in current cultural, social and activity systems it is not possible to define a single definition of musical creativity. On the contrary, she argues for “the broadening of the concept of “musical creativity” to include a plurality of equally valid creativities through which musicians may fluidly move or situate within realms of creating and receiving musical artworks and cultural products.” Among which she mentions, e.g., individual creativity, collaborative (group) creativity, communal creativity, emphatic creativity, performance creativity and others (p. 15-16). Cook (2011) used a similar argument by pointing out that the term refers to “an indefinite number of related concepts or behaviors.” Cook suggests that musical creativity “revolves round social interaction, and is embedded and embodied in the practices of everyday life” (p. 451). According to Hargreaves (2012), musical creativity is “only one facet of a much broader phenomenon, the central core of which is imagination, which incorporates creative perception as well as production.”

At conception of our study, we have not found a musical creativity scale or assessment that would, in our view, adequately capture the musical behavioral changes induced by the DHP. The lack of objective creativity measurement has been clearly admitted in the Limitations section of the Discussion.

The behavioral outcomes of DHP workshops have thus been evaluated with detailed structured questionnaires (18 questions), including one question about perceived change in musical creativity and another about change in broadening the perception of sounds from the environment (“opening the ears”) after taking the course.

The behavioral observations from the previous DHP workshops (Synek, 2008; Medek et al., 2014) have inspired the hypotheses of the present study, both the expected behavioral change and the neuroimaging correlates.

### Behavioral Results

Behavioral results showed changes in esthetic/emotional perception, predominantly of New music and non-musical sounds, possibly induced by the DHP training. Increased music liking has been described after repeated exposure (McDermott, 2012), however, this cannot be the sole explanation of our results. First, sound samples were randomly selected for each sound class each time, so the subjects would not hear exactly the same selections in sessions 1 and 2, even though some repetitions may have occurred. Second, and more importantly, repetition effects would manifest in both groups, whereas most increases in the “like” scores happened in the active (DHP training) group. The only statistically increase in the control group (uncorrected) appeared for Classical music, which, interestingly, was far away from achieving significance in the Active group. However, this effect and the inter-group discrepancy may be due to a ceiling effect, since both groups’ likings were close to 100% (see Table 1). The DHP sounds may present a special case for the active group. The DHP course by definition exposed the participants to many different sounds from the same DHP sound class, even if not identical to those presented during the fMRI examination. Perhaps they were similar enough to increase the liking by repetition, a marked change, which was statistically most robust and significant, even after Bonferroni correction. The composing process itself is important as well, when the active group had an experience with producing/creating sounds and creating sound compositions related to DHP and New Music samples. The DHP group while composing uses pre-recorded environmental sounds, whether nature or industrial, as well. These, however, represent only a portion of the DHP “elements,” which would explain why the change in the active group was smaller and less robust (only significant without Bonferroni correction). One might also argue that environmental sounds would be very familiar to the adult participants and thus not likely to experience liking change by repetition during the DHP course. Here, we also consider that during the DHP course, they are used with motivation and purpose (not just passive listening), which may support the observed increase in liking. Finally, the New music samples have certain similarity to all three mentioned sound classes (DHP, Nature, Industrial), so again, taking the DHP course (composing, improvising, listening and performing activities) may influence New music perception in a similar way as actual repeated hearing New music samples. The increase in New music liking, like that for Nature and Industrial, is smaller and less robust than for the DHP samples, as would be expected.

As for the necessity of Bonferroni correction, we suggest that it depends on the perspective of the data. If we assumed that listening to each sound class was an independent process, generating a separate dependent liking variable, then we believe Bonferroni correction would not be required, as we were not performing repeated testing on the same data. If, however, we take liking as one common dependent variable and the 5 particular sound classes and 2 sessions as mere instances, then a correction would be necessary.

### Imaging Results

Our hypothesis was that DHP training with composing music would change the brain activation as a functional correlate of enhancement of general creativity, i.e., widening of participants’ possibilities/flexibility to incorporate new sounds as elements of composition of music. Our imaging findings suggest that DHP training modified the response to diverse sound samples, differentially changing the engagement of functional networks known to be related to domain-general creative thinking, namely, decreasing DMN deactivation and decreasing activation of EN (see, e.g., Beaty et al., 2017).

In studies of neural correlates underlying the musical creativity, researchers have been investigating brain activity during novel music creation, as Bengtsson et al. (2007) mentioned “improvisation arguably satisfies the demands of a prototypical creative behavior.” For example, Limb and Braun (2008) suggested in their fMRI study that dissociated pattern of activity in medial and lateral prefrontal cortices is associated with the neural substrate of improvisation and spontaneous musical creativity. Furthermore, in a meta-analysis (Boccia et al., 2015) of the fMRI studies in three domains of creativity, i.e., musical, verbal and visuo-spatial, revealed that musical creativity is associated with activations in bilateral medial frontal gyrus, in the left cingulate gyrus, middle frontal gyrus (MFG), and IPL and in the right postcentral and fusiform gyri, while the general meta-analysis in all the three domains showed the activated clusters in the bilateral occipital, parietal, frontal, and temporal lobes.

However, the comparison of our results with the studies involving active creative process is not straightforward as we investigated effects of the creativity training during passive listening task with subsequent sample evaluation. Hence, in the following sections, we primarily discuss our results according to distinct functional systems that were modulated by the training intervention, followed by interpretation of the results in the context of the neural correlates of creativity.

#### Auditory and Motor Networks

Primarily, regions in the auditory network and motor networks were expected to be activated during our fMRI measurements, as functional neuroimaging studies document these two networks interacting during auditory perception as well as music production (Chen et al., 2007; Zatorre et al., 2007). There are many recent studies that investigated brain activation during music/sound/speech sample perception (Hanke et al., 2014; Hong and Santosa, 2016; Casey, 2017; for review in music perception, see Angulo-Perkins and Concha, 2014; Janata, 2015). The motor cortical areas are considered to play an important role in perception of temporal patterns during music listening, as a meta-analysis of the studies of perception rhythmic patterns suggested common activations of premotor areas (Janata and Grafton, 2003). Especially, activation in pre-SMA and SMA have been observed during beat perception (together with premotor cortex, basal ganglia and cerebellum) (Grahn and Brett, 2007), temporal perception (together with in ACC) (Pastor et al., 2006), and during distinguishing changes of rhythmical features (together with the premotor area) (Popescu et al., 2004). Activation in several areas in motor network can also be observed during music improvisation, possibly functioning as sequence processing centers: pre-SMA and dorsal premotor cortex (dPMC) (de Manzano and Ullén, 2012; Donnay et al., 2014); pre-SMA, dPMC as well as DLPFC, and the left posterior part of the STG (improvise > reproduce) (Bengtsson et al., 2009); and the dPMC, ventral PMC, together with areas in EN (IFG and ACC) in a different study (improvisation > playing patterns) (Berkowitz and Ansari, 2008).

In our model, activation changes in regions of the auditory network (i.e., decreased activation in right STG, PT, TP, right MTG, and enhanced deactivation in left MTG) and the motor network [i.e., deactivation in bilateral superior frontal gyrus [SFG] (pre-SMA and SMA), and decreased activation in the left cerebellum, right precentral gyrus [preCG] (dPMC)] are observed in the DHP trained group, whereas activation in these networks was increased in the control group. The strengthening with repetition in the control group can be regarded as a priming effect, engaging both the “listening” network and the “music-making” network, a learning effect which may be expected. On the other hand, the weakening of the response in the active group may come as a surprise, especially since the group has been engaged in 2-day group composition and performance, employing both the auditory and motor networks. Perhaps the weaker response in the “modality-specific” networks actually reflects a change of balance toward “modality-independent” networks engaged in music perception and creation, as a result of the DHP training. Previously, activation decrease with repetition was reported, so-called repetition suppression (e.g., Brown et al., 2013), however, the effect was observed in short-term within-session studies, not across sessions. Also, our study design did not involve exact repetition, as our sound samples were randomly selected and ordered. Our longitudinal design including a control group permits separation of general time and repetition effects from training-induced plasticity (Olszewska et al., 2021).

#### Default Mode and Executive Networks

Next, as a result of the *post hoc* analysis, we observed significant loss of task-related deactivation in PCC and precuneus in the second session in the active (DHP-trained) group. These areas are the main hubs of the DMN (Raichle et al., 2001), which was originally known to be deactivated during focused cognitive task performance and oppositely activated during the rest/mind-wondering. Whereas cognitive tasks requiring focused attention deactivate the DMN, DMN apparently becomes active during internally focused tasks, spontaneous cognition and/or broad awareness of the environment (Raichle et al., 2001; Buckner et al., 2008; Andrews-Hanna et al., 2010). For our data, it is tempting to consider the observed “loss of task-related deactivation” in the DMN as “broadening” of the attention during listening to the sound samples after training, in parallel to the above mentioned behavioral “widening” of the inner concept of (pleasant) music.

In previous studies in creativity, several researchers suggested relationship between creativity and DMN (Jung et al., 2010; Takeuchi et al., 2011, 2012; Wei et al., 2014; Bashwiner et al., 2016; Fink et al., 2018). For example, Takeuchi et al. (2011) observed that reduced task-related deactivation in the precuneus was associated with higher individual domain-general creativity, where the relationship between creativity and brain activity during working memory task was investigated. In these studies, creativity was assessed using divergent thinking (DT) tests (Guilford, 1967), which have been recognized as indicators of creative ability and often been used in neuroscientific studies of creativity due to their relationship to flexibility, fluency and originality, since psychologists have demonstrated these characteristics associated with highly creative people (Guilford, 1950; Drevdahl, 1956). The neurobiological correlates of improvement in those individual DT components (flexibility, fluency, originality) were evaluated in three neuroimaging studies of domain-general creativity training (Wei et al., 2014; Fink et al., 2015; Sun et al., 2016). Wei et al. (2014) especially observed positive correlation of originality with the strength of resting-state functional connectivity between medial prefrontal cortex (mPFC, a hub node of DMN) and MTG, whereas other two studies showed improvement of fluency and originality associated either with increased activation in the left IPL and decreased activation in the left MTG (Fink et al., 2015), or with increased activation in the bilateral DLPFC, dACC, right precuneus and left IPL (Sun et al., 2016). Since the DHP method of group composing using non-traditional instruments and environmental “non-musical” sounds is closer to DT training than domain-specific musical training such as musical improvisation, the functional changes observed in the DMN in this study may, similarly, reflect improvement in the originality and fluency components of domain-general creativity. However, such interpretation of the loss of deactivation in PCC and precuneus would be speculative since the previous changes in DMN were located elsewhere (mPFC and IPL). Furthermore, no direct assessment of DT components was performed in our study since the DHP had been originally developed independent of the concept of DT.

Structural MRI studies have also demonstrated association between creativity and gray matter change in the DMN. Jung et al. (2010) observed a positive correlation between high domain-general creativity and regional cortical volume and thickness of the right PCC, right angular gyrus (AG) and lower left lateral OFC, whereas a study by Bashwiner et al. (2016) showed that high musical creativity correlated with increased cortical surface area or volume not only in the regions associated with motor activity and sound processing, but also in three out of four nodes of the DMN (i.e., dorsomedial PFC, lateral temporal cortex, and TP).

In our study, greater deactivation in the right ACC, left OFC/IFG, AG and decreased activation in the right posterior MFG and supramarginal gyrus (SMG)/AG (IPL) was also observed in the second session in the active group. These areas constitute the EN (Seeley et al., 2007), which has been associated with working memory, problem solving, and decision making. Interestingly, a fMRI study revealed negative association with total hours of improvisation experience and activation in the EN during musical improvisation by professional musicians compared to rest (Pinho et al., 2014), while another study showed greater deactivation of EN during the generation phase of new poetry in experts compared to that in novices (Liu et al., 2015). With respect to the salience network, the observed training-related decreases in the right-sided ACC and bilateral insula (see Table 2) may, in fact, reflect decreased activation in the salience network.

Existing studies suggest a relationship between creativity and activity patterns engaging both DMN and EN in a specific manner. For example, Beaty et al. (2015) observed functional networks consisting of regions within the DMN (PCC, precuneus, and IPL) and EN (DLPFC) changing their coupling according to the current stage of the DT task performance in temporal (time-resolved) connectivity analysis. Similarly, during poetry composition, in which generation and revision phases were separated, Liu et al. (2015) observed that a hub region of DMN (mPFC) was activated during both phases, whereas activation in EN (DLPFC and IPS) was increased only during the revision phase. Also, in a study by Marron et al. (2018), the subjects with higher-creative potential (assessed by scores in the DT task) showed greater activation in the DMN as well as reduced activation in the EN during the task, compared to the lower-creative potential groups.

Even though the above studies investigated brain networks engaged in creative tasks, whereas our active group passively listened to sound samples, apparently, there was a common observation of greater DMN activation (or less DMN deactivation) together with decreased EN activation (or greater EN deactivation). We may therefore speculate that the DHP training modulates these brain networks in the same way as domain-general creativity training.

Taken together, our results from the imaging analysis may suggest that the participants of DHP were “differently hearing” sound samples in the second session. Potentially, this could mean that they were not only passively hearing but also actively perceiving sounds as materials/elements of music, as in a pre-stage (thinking phase) of composing music. In such case, DHP training could possibly widen/enhance the flexibility of subjects’ music perception toward non-musical sounds, in the similar way of the concept of divergent thinking. Of course, this conclusion would be much stronger if it were supported by specific behavioral data, as well as by sound-class specific training effects in the active group (i.e., group by time by sound class interaction), which was not found in the present data.

### Limitations

Neither cognitive tests of creativity nor in-scanner task of creativity were performed. Thus, our study was mainly focused on the “wondering phase” but not “producing process.” The results of such testing, if compared with the task performed here focusing on the “wondering phase,” could provide further validation of our conclusions and shed more light on the “producing process.”

Choice of musical/non-musical sound samples did not systematically cover all music genres (e.g., There were no samples from Pop, Rock or electric music representative as more popular musical sounds among young people, instead, some sound compositions were quite similar between the samples of New music class and DHP).

Furthermore, since the control group only experienced normal student activities without any control/sham training and only the active group underwent an intervention, the possibility of “placebo” effect (e.g., due to subconscious expectations) in the active group could not be completely ruled out.

Finally, although we recognize greater potential for plasticity of the children’s brains, we opted for recruitment of university students due to methodological and ethical limitations of neuroimaging in under-age subjects. Still, previous data suggested that behavioral data of DHP training are similar in the young adults as in children.

### Future Directions

Because of previous studies suggesting association between creativity and functional connectivity within/between brain networks such as DMN/EN, we plan to analyze functional connectivity changes induced by DHP training.

We also plan to further explore the brain responses of the active group to Natural sounds. Individual responses in the active group showed lower brain activation for the class of Natural sounds (stream, birds, and rain, etc.) compared to all other sound classes. If this variable phenomenon were to be confirmed as a statistically significant group effect, this type of sound could be used in educational, artistic and therapeutic activities.

## Conclusion

We suggest that DHP seems effective to broaden sound and music preferences and perception, as reflected in both behavior and brain function. Neuroanatomical location and character of the training-induced changes suggest their possible relationship to domain-general creative processes.

## Data Availability Statement

The raw data supporting the conclusions of this article will be made available by the authors, without undue reservation.

## Ethics Statement

The studies involving human participants were reviewed and approved by Ethics Committee of the Faculty of Education at Palacký University Olomouc. The participants provided their written informed consent to participate in this study.

## Author Contributions

PHl and VZ conceived the study. GV, PHl, VZ, and AA performed the literature search. VZ, GV, GC, and JS recruited the participants and trained them for the experiments, PHl, VZ, GV, GC, JS, and PHo designed the fMRI experiment, prepared the stimuli, and supervised data acquisition. PHo and PHl designed the fMRI analysis. JV and MT designed and performed the statistical analysis of behavioral data. AA, PHo, and PHl drafted the manuscript. All authors have participated in interpretation of the results and critical revisions of the manuscript.

## Conflict of Interest

The authors declare that the research was conducted in the absence of any commercial or financial relationships that could be construed as a potential conflict of interest.

## Publisher’s Note

All claims expressed in this article are solely those of the authors and do not necessarily represent those of their affiliated organizations, or those of the publisher, the editors and the reviewers. Any product that may be evaluated in this article, or claim that may be made by its manufacturer, is not guaranteed or endorsed by the publisher.

## Footnotes

1
www.audacityteam.org
